# A Longitudinal Mixed Methods Case Study Investigation of the Academic, Athletic, Psychosocial and Psychological Impacts of Being a Sport School Student Athlete

**DOI:** 10.1007/s40279-024-02021-4

**Published:** 2024-04-18

**Authors:** Ffion Thompson, Fieke Rongen, Ian Cowburn, Kevin Till

**Affiliations:** 1 Room G07, Cavendish Hall, Carnegie School of Sport, Leeds Beckett University, Headingley Campus, Leeds, LS6 3QS UK; 2 Queen Ethelburga’s College, York, UK; 3 School of Science and Technology, Nottingham Trent University, Nottingham, UK; 4 Leeds Rhinos Rugby League Club, Leeds, UK

## Abstract

**Background:**

Sport schools are popular environments for simultaneously delivering education and sport to young people. Previous research suggests sport school involvement to have impact (i.e. the positive/negative, intended/unintended and long/short-term outcomes, results and effects) on student athlete’s holistic (i.e. academic, athletic, psychosocial and psychological) development. However, previous research is limited by (1) cross-sectional methods, (2) limited multidimensional assessments, (3) lack of consideration for athlete characteristics (e.g. sex) and (4) failure to evaluate how sport school features affect student-athlete impacts.

**Objectives:**

The study, using a mixed methods case study approach, aims to (1) longitudinally evaluate the impact of sport school involvement on the holistic development of student athletes, (2) evaluate the impact on holistic development by student-athlete characteristics and (3) explore the features and processes of the sport–school programme that drive/facilitate holistic impacts.

**Methods:**

A longitudinal mixed methods design was employed across one full academic school year (33 weeks). Six data-collection methods (i.e. online questionnaire, physical fitness testing battery, academic assessment grades, log diaries, field notes/observation and timeline diagram/illustration) were used to assess the academic, athletic, psychosocial and psychological impacts for 72 student athletes from one sport school in the United Kingdom (UK).

**Results:**

Student athletes developed positive long-term holistic overall impacts (i.e. academically, athletically and personally), including maintaining stable and relatively high levels of sport confidence, academic motivation, general recovery, life skills, resilience and friends, family and free time scores. Despite positive impacts, juggling academic and sport workload posed challenges for student athletes, having the potential to lead to negative holistic impacts (e.g. fatigue, stress and injury). Positive and negative impacts were linked to many potential features and processes of the sport school (e.g. academic and athletic support services versus insufficient training load build-up, communication, coordination, flexibility and planning). Furthermore, when considering student-athlete characteristics, females had lower sport confidence, higher general stress and body image concerns and less general recovery than males and student athletes who played sport outside the school had lower general recovery.

**Conclusions:**

This mixed method, longitudinal study demonstrated sport school involvement resulted in many positive academic (e.g. good grades), athletic (e.g. fitness development), psychosocial (e.g. enhanced confidence) and psychological (e.g. improved resilience) impacts attributed to the academic and athletic support services provided. However, juggling heavy academic and athletic workloads posed challenges leading to negative impacts including fatigue, pressure, stress and injury. Furthermore, holistic impacts may be sex dependent and further support may be required for female student athletes in sport school environments. Overall, these findings demonstrate the complex nature of combining education and sport commitments and how sport schools should manage, monitor and evaluate the features of their programme to maximise the holistic impacts of sport–school student athletes.

## Key Points

**Table:** 

Sport–school involvement resulted in many positive academic (e.g. good grades), athletic (e.g. fitness development), psychosocial (e.g. enhanced confidence) and psychological (e.g. improved resilience) impacts attributed to the academic and athletic support services provided. However, juggling heavy academic and athletic workloads posed challenges leading to negative impacts including fatigue, pressure, stress and injury.
Biological sex and external sport commitments were shown to influence student-athlete holistic impacts. Females had lower sport confidence, higher general stress and body image concerns and less general recovery than males. Student athletes who played sport outside the school had lower general recovery.
Positive impacts were attributed to the academic and athletic support services provided and personal traits of the student athletes and staff. Negative impacts were associated with insufficient training load build-up, communication, coordination, flexibility and planning.

## Introduction

In response to the potential negative consequences associated with the intensification of youth sports programmes [e.g. 1, 2] and the drive for a more holistic approach to youth athlete development [e.g. 3], there has been a cultural shift towards combining sport and education in supportive environments to appropriately prepare individuals for working life if they do not become professional athletes [4]. This type of approach is referred to as a ‘dual career’ (DC) approach (i.e. combining sporting pursuits alongside education or vocational endeavours). A DC approach has long been evident in the USA, where collegiate athletes pursue university education alongside elite performance in Olympic sports or before entering the draft system for professional sports (Ryba et al. 2015). However, it has recently become more prominent in the United Kingdom (UK, [5]). Morris et al. [6] further distinguishes between different dual career development environments (DCDEs; i.e. environments that support DC approaches) based on the different structures and approaches used to provide both athlete development and academic support.

One example of a DCDE that aims to cater for youth athletes’ holistic development is a sports school. Sport schools are a key environment for DC development in many countries and are considered an increasingly integral part of a nations’ elite sport performance strategy [7]. Sport schools aim to combine sport and education to offer student athletes considerable academic flexibility (e.g. adaptation of school and training schedules and lighter load by one subject) and athletic support (e.g. high-quality coaches and physiotherapy) [8]. Recently, Morris et al. [6] categorised two types of sport schools: sport-friendly and elite. Both sport-friendly schools and elite sport schools are situated in lower and upper general and vocational secondary education (i.e. International Standard Classification of Education level 2–5). However, unlike a sport-friendly school, an elite sport school has formal communication with a sport federation, often receiving funding [6].

While a DC approach holds promise for enhancing the development of school-aged athletes, it brings forth various potential challenges. These challenges include managing academic study and training alongside competition schedules, dealing with fatigue/lack of sleep and being forced to make personal sacrifices [9–11]. Consequently, despite the intention of sport schools to provide a platform for athletes to balance sport and education, the reality is that they introduce heightened demands, potentially subjecting student athletes to risks of burnout and injury, as identified in previous research on intensified youth sports (e.g. [1, 12]).

The process of youth athletic development within a school is complex, as athletes experience psychological, physical and psychosocial growth in an environment where they are navigating competing sport, academic and social demands [13]. Consequently, sport school involvement will impact (i.e. the positive/negative, intended/unintended and long/short-term outcomes, results and effects) an individual’s holistic development across academic, athletic, psychosocial and psychological dimensions [3, 14]. Recognizing the diverse and extensive potential impacts of DCDEs (such as sport schools), aligns with the overarching idea of examining student athletes holistically. This comprehensive perspective is vital in understanding and navigating the multifaceted impacts of sport schools on the developmental trajectory of individuals [3, 14].

Increasingly, research has explored such impacts on holistic athlete development. A recent mixed methods systematic review [8] highlighted there are a multitude of immediate, short- and long-term positive (e.g. physical development, more stable levels of general health and well-being, status/popularity and life skills) and negative (e.g. lower higher education attainment, limited experience with ordinary life outside of competitive sport, high number of injuries and performance pressure) impacts associated with the athletic, academic, psychosocial and psychological development of sport school student athletes. However, this systematic review identified several limitations within the current evidence base, including: (1) limited research examining how sport-friendly school features are operationalised in different contexts (e.g. UK), (2) a failure to evaluate multi-dimensional domains of athlete impact, often focussing on one or two dimensions and (3) limited research evaluating how features affect athlete impacts (i.e. causal relationship between the characteristics and features of sport school and holistic athlete impacts).

Subsequently, two studies [15, 16] assessed the impacts of a UK sport-friendly school on student athletes across all four domains of holistic athlete development (i.e. academic, athletic, psychosocial and psychological). Overall, the findings of both studies demonstrated a multitude of positive impacts associated with being a sport school student athlete but, also, impacts of concern. However, both studies were cross-sectional in nature (i.e. use of a single moment of measurement), where exposure and impacts were simultaneous. Consequently, these studies oppose the nature of ‘transition’ as a process and the dynamic nature of sport-friendly school environments. Therefore, longitudinal research designs are required to investigate student-athlete development or changes over time. Additionally, although Thompson et al. [15, 16] provided a general overview of the features and multiple possible impacts of sport school involvement, it is important to note that not every athlete experienced every potential impact. Instead, impacts varied across individuals and were driven by their individual characteristics and experiences of sport school features over time. Sport schools would benefit from an approach that is aware of individual differences and how they may impact a student athlete’s journey. Accordingly, it is important to explore the specificity of athlete characteristics/variables (e.g. biological sex) as holistic impacts may vary considerably depending upon an athlete's sex, sport requirements and boarding status [17–19].

Finally, given the complex and dynamic nature of DC environments [20], where student athletes have to interact with coaches, programme culture and practices, research needs to explore the features and processes (i.e. the context-individual interactions) of sport-friendly school programmes that drive and facilitate positive and negative holistic impacts described by Thompson and colleagues [15, 16]. Moreover, within the UK, there are substantially more sport-friendly schools, with only one identified example of an elite sport school found in Scotland [5]. Sport-friendly schools in the UK tend to be more independent than the systemic approach in other countries (e.g. Germany and Sweden [21]). In the UK, the development of a sport-friendly school is primarily a matter for individual schools and is often pursued as part of a strategy to create a distinct identity. As a result, it is important to investigate the individual context of a sport-friendly school within the UK as a case study.

Based on the above, this study, using a mixed methods longitudinal case study design, aims to (1) longitudinally evaluate the impact of sport-friendly school involvement on the holistic (i.e. academic, athletic, psychological and psycho-social) development of student athletes, (2) evaluate the impact on holistic development by athlete characteristics (i.e. sex, boarding status and external sport involvement) and (3) explore the features and processes of the sport-friendly school programme that drive/facilitate positive and negative holistic impacts.

## Methods

### Research Approach

This study was aligned with and guided by a critical realist (CR) perspective. In line with North’s [22] perspective on CR, this study was guided by the principles of developing theory (i.e. first understanding of sport schools impacts, then, second, developing an understanding of ‘how,’ ‘why’, ‘what’ and ‘for whom’). As such, the researcher first engaged in contextual description (aims 1 and 2), then, second, started to develop an understanding/explanation of how observed patterns were generated (aim 3). To help achieve the study aims, this study adopted a concurrent mixed methods approach (i.e. qualitative and quantitative data collected simultaneously [23]). This design aims to create mutually exclusive sets of data that inform each other [24]. Furthermore, the qualitative and quantitative data were analysed separately but then integrated to cross-validate findings. Finally, in line with the CR stance of establishing ‘how’, ‘why’, ‘what’ and ‘for whom’, Pawson and Tilley's [25] and Yin’s [26] guiding principles for an explorative case study approach were used.

### Positionality of the Researchers

It is also important to acknowledge the collective roles of the researchers’ autobiographies, values and beliefs in describing, designing and interpreting the findings [27]. To acknowledge this, we consciously outline them to help appreciate and evaluate the results in nuanced ways [28]. The first author, F.T., collected the data and was lead on the analysis and writing. As the school’s lead strength and conditioning (SC) coach and a previous student athlete at a different sport-friendly school for 5 years, this would have inevitably shaped the primary researchers’ conceptions and influenced the study’s initial framing, design and analysis. Furthermore, the collective experiences of the remainder of the research team will have contributed to the interpretation of the data and shaping of the results. Combined, K.T., F.R. and I.C. have over 30 years of research and applied experience within athlete development systems.

### Context of Study

One sport-friendly school (pseudonym ‘Nunwick High’) was selected for the study based on Morris et al.’s [6] definition of a sport-friendly school. The selection of ‘Nunwick High’ was information-oriented and opportunistic. ‘Nunwick High’ has 8 years of experience providing DC support through a performance sport pathway embedded within a UK independent school. ‘Nunwick High’ has eight performance sports as part of its performance programmes: athletics, basketball, cricket, football, hockey, netball, rugby and swimming, targeted at year groups 7–13 (aged 12–18 years). Each student athlete enrolled on ‘Nunwick High’ performance sport programme receives a place to study, train and, in some cases, live during their lower and upper secondary school years, including access to learning facilities, a sport science centre, a sport treatment centre, sport facilities, accommodation buildings and a canteen all in one proximity (single campus). Based on the information above, ‘Nunwick High’ represented an established and mature environment that should be a rich source of information.

### Participants

Participants had to meet the following inclusion criteria: participate as a student athlete in one of the performance sport programmes within ‘Nunwick High’ and be aged 16 or above (years 12–13). Years 12–13 were chosen specifically, as during this stage student athletes are transitioning to a more intense and structured period of athletic development [29, 30], and increased educational demands, with the consequence that the management of their DC, is a distinct concern. A total of 72 student athletes (mean age 17.29 ± 0.52 years, 48 male and 24 female) participated in the study. At baseline (T1) the student athletes had been attending and competing at ‘Nunwick High’ for an average of 1.2 ± 1.5 years (range from 2 weeks to 7 years). Out of the 72 student athletes, 31 were boarders (i.e. live at the school) and 41 were non-boarders, 31 played sport externally to the sport-friendly school and 41 only played sport for the sport-friendly school, representing the following sport: athletics (*n* = 4), cricket (*n* = 4), hockey (*n* = 12), netball (*n* = 9), football (*n* = 18), rugby (*n* = 15) and basketball (*n* = 10).

### Study Design

A longitudinal mixed methods case study design was employed across one full academic school year (33 weeks). To engage in a comprehensive and holistic investigation of the impacts of being a sport-friendly school student athlete and the features and processes that drive/facilitate such impacts, six data-collection methods were utilised: (1) online questionnaire, (2) physical fitness testing battery, (3) academic assessment grades, (4) log diaries, (5) field notes/observation and (6) timeline diagram/illustration.

**Fig. 1:**
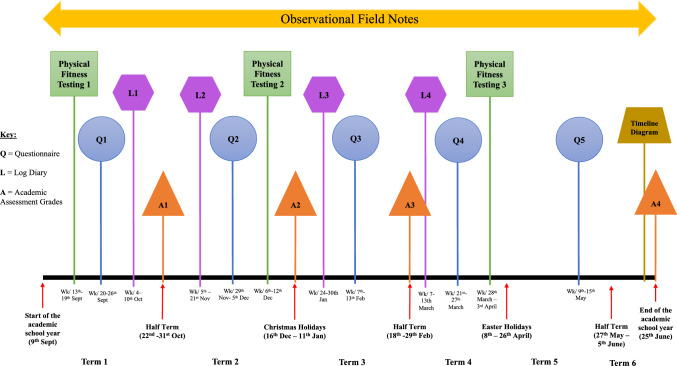
Overview of data collection points at the sport-friendly school

The online questionnaire occurred over five data collection periods (Q1, September; Q2, November/December; Q3, February; Q4, March; and Q5, May). The physical fitness testing battery occurred over three data collection periods (PFT1, September; PFT2, December; and PFT3, March/April). The academic assessment grades occurred across four data collection periods (A1, October; A2, December; A3, February; and A4, June). The log diary occurred over four data collection periods (L1, October; L2, December; L3, January; and L5, March). The observational research was ongoing throughout the whole academic year (33 weeks). Finally, the timeline diagram/illustration was collected once at the end of the academic year. Figure 1 provides an overview of the data collection timeline. The university sub-ethics committee granted this study (ref. 86728) with online informed assent and parental written consent obtained.

### Measures

#### Online Questionnaire

Data collection involved participants completing an online questionnaire (predicted completion time, 29 min) that provided a multi-dimensional assessment of holistic athlete impacts identified in previous literature [8, 15, 16]. The online questionnaire comprised of 12 domains (i.e. academic and sport workload, difficulty balancing sport and academics, academic support and satisfaction, injury and illness, rest and recovery, body image, family, free time and friends; sport competence; sport confidence; life skills, dual career motivation and resilience) as presented in Table 1. The questionnaire was conducted in a quiet room, and student athletes were allowed sufficient breaks when required and were allowed to return to the questionnaire at a later time within the same day. Further, open-ended questions were used to help expand on responses to close-ended questions [31], providing further information on the features and processes that drove/facilitated specific impacts. All questionnaires were collected across all timepoints (T1–T5) apart from The Life Skills Scale for Sport (LSSS) questionnaire which was added in from T2 as the LSSS requires participants to rate how much their environmental exposure has taught them to perform the skills listed within the questionnaire and a baseline value was not appropriate. Completion rates: 97% for Q1, 90% for Q2, 94%for Q3, 93% for Q4 and 99% Q5.

**Table 1:** Summary of online questionnaire administered to provide a multi-dimensional assessment of holistic athlete impacts

Holistic domain	Sub-domain	Questionnaire	Explanation
Sport and Academic	Academic and sports workload	Custom questionnaire	Informed by [32, 33], athletes were asked on a 13-item slider scale to quantify over the last two months a weekly average for: (1) hours doing extra-curricular activities, (2) hours spent in academic lessons, (3) hours doing home-work, (4) number of academic lessons missed due to sport, (5) hours of external training hours, (6) number of external competitions, (7) number of external SC sessions, (8) hours of internal training hours, (9) number of internal competitions, (10) number of internal SC sessions, (11) number of performance analysis sessions, (12) number of recovery/rehab sessions and (13) number of rest days
	Difficulty balancing academic and sport workload	Custom questionnaire	Student athletes were asked to rate on a 1-item five-point Likert scale (ranging from extremely difficult to extremely easy) how difficult they were currently finding balancing the academic work and sport There was an open-ended text box, where student athletes were able to provide a rationale and expand on the reason for their answer to how difficult they were finding balancing academic and sport workload
	Academic Support and Satisfaction	Custom questionnaire	Student athletes were asked to rate on a 1-item five-point Likert scale (ranging from extremely dissatisfied to extremely satisfied) how satisfied they were with the academic support they were currently receiving (one item) To further support this, there was an open-ended text box, where student athletes were able to provide a rationale and expand on the reason for their answer
	Dual career motivation	The European Student-Athletes’ Motivation Towards Sports and Academics Questionnaire (SAMSAQ-EU; [34, 35])	The 40-item questionnaire has been shown to being suitable for use within youth populations [e.g., [36] Participants were required to indicate their level of agreement (i.e., from a minimum of 1—very strongly disagree to a maximum of 6 —very strongly agree) to each SAMSAQ-EU item [34, 37, 38] The SAMSAQ-EU questionnaire was calculated based on Lupo et al. [35] federation three-factor model (SAM,12 items; AM, 13 items; and CAM, 5 items), as this represented student-athletes classified according to the UK dual career policy
Athletic/physical	Injury and illness	Custom questionnaire	Across 5-items, student athletes were asked in the 2 months prior to the questionnaire, regardless of the consequences for participation in regular training and/or competition: (1) if they had sustained an injury (yes/no), (2) state what injury they had sustained (open ended text entry), (3) how ‘Nunwick High’ had dealt, adapted and supported them with the injury (open ended text entry), (4) how many training sessions they had missed or had adapted due to their injury (slide scale) and (5) how many academic lessons they had missed due to their injury (slide scale) The same questions were then repeated but with regards to illness (not injury)
	Rest and recovery	Sport Recovery-Stress Questionnaire (RESTQ-52-Sport; [39])	The RESTQ-52 sport has good construct validity and good-to-satisfactory internal reliability [39]. In addition, previous research has shown suitability for use with youth populations (e.g. [40] and [41] Participants were asked to respond to the 52-items on a seven-point Likert-type scale anchored by descriptors ranging from ‘never’ (0) to ‘Always’ (6), indicating how much the statements applied to them over the last 2 months Nineteen subscale scores were derived, which were further grouped into the following four major subscale groups: (1) general stress subscale, 2) general recovery activity subscale, 3) sport-specific stress subscale and 4) sport-specific recovery activity subscale The four major subscales were scored by calculating a mean of the sub-items with a maximum score of 6
	Sport competence	Sport Competence Inventory (SCI; [42])	The questionnaire has previously demonstrated adequate psychometric properties, reliability and validity, in addition to being suitable for use within youth populations [42] Across three items, participants rated their competence in technical, tactical and physical skills on a 5-point scale ranging from ‘not at all competent’ to ‘extremely competent’ Sport competence was scored by calculating a mean of the three items with a maximum score of 5
	Sport confidence	The self-confidence subscale of the Revised Competitive State Anxiety-2 (CSAI-2R; [43])	Previous research has shown suitability for use with youth populations (e.g. [44]) and established good factorial validity [43] This measure is composed of five items rated on a 4-point scale ranging from ‘not at all’ to ‘very much so’ Sport confidence is scored by calculating a mean of the five items with a maximum score of 4
Psychosocial and psychological	Body image	The Eating Attitudes Test (EAT-26; [45])	The 26-item version is highly reliable and valid [45–47] and has been used previously to assess ‘eating disorder risk’ in sports schools, high schools, colleges and other unique risk samples such as athletes [48–50] Items are presented in a 6-point Likert scale ranging from 1 (‘never’) to 6 (‘always’) An EAT score is calculate by adding the scores for each item together for a total score In the current study, subjects scoring EAT 0–9 are defined as subjects with normal eating behaviour attitudes and EAT ≥ 10 as subjects with disordered eating behaviour and attitude [51, 52]
	Family, free time and friends	The autonomy, parent, peer and social support sub-scales from the KIDSCREEN-27 Health Questionnaire for Children and Young People [53]	Suitable for children and adolescents aged 8–18 years [54]. All domains have reported satisfactory-to-good internal consistency and a 2 week test reliability was deemed adequate for all dimensions [54] For this study, the degree of satisfaction with financial resources was removed Participants were asked to answer ten items regarding the last 2 months on a 5-point Likert-type scale (‘never, seldom, quite often, very often and always’) An open-ended question was added where student-athletes could elaborate on how the features of ‘Nunwick High’ had influenced their answers to the questionnaire For the peer and social support sub-scales scores were converted to Rasch person parameter estimates and then into *T*-values, using the SPSS syntax provided resulting in subscale scores with a scale mean around 50 and standard deviation around 10, with higher values indicating, higher HRQoL [53] For the parent relations and autonomy sub-scales scores were calculated by summing the score of the five sub-items with a maximum score of 25
	Life skills	Life Skills Scale for Sport (LSSS [55])	Previous research provided evidence for the validity and reliability of this 43-item version scale with youth sports participants [55] Participants were asked to ‘rate how much you perform the skills listed below.’ Responses were provided on a 5-point scale ranging from 1 (‘not at all’) to 5 (‘very much’) Example items included: teamwork (seven items), goal setting (seven items), time management (four items), emotional skills (four items), interpersonal communication (four items), social skills (five items), leadership (eight items) and problem solving and decision making (four items) An overall LSSS score is scored by calculating a mean of the sub-items with a maximum score of 5
	Resilience	The Brief Resilience Scale (BRS; [56])	The BRS has shown high test–retest reliability and validity [56], in addition to being suitable for use within youth populations (e.g. [57]) The scale consists of six items that measure the ability to recover after stress rated on a 5‐point Likert scale (1, strongly disagree; 5, strongly agree) The BRS is scored by calculating a mean of the six items with maximum score of 6

See Thompson et al. [16] for further details

#### Academic Assessments Grades

To assess educational attainment, termly academic subject assessment grades were extracted from the school administrative system. As all student athletes were in years 12–13, and grades were provided in the UK national curriculum grading format for Advanced level (A-level) and Business and Technology Education Council (BTEC) qualifications. To adequately compare BTEC and A-level grades, in addition to statistical purposes, academic assessment grades were converted to a number using a school grades translation matrix in Table 2 (similar to [58]). After conversion, an average of each individual’s subject score was calculated to get one overall academic assessment score for each student athletes. Completion rates: 96% for A1, 90% for A2, 94% for A3 and 94% for A4.

**Table 2:** School grades translation matrix

A-level assessment grade	BTEC assessment grade	Awarded score
A^a^	Distinction^a^	6
A	Distinction	5
B	Merit	4
C	Pass	3
D	–	2
E	–	1
F	Fail	0

^a^Star; A-level, advanced level; BTEC, Business and Technology Education Council

#### Physical Fitness Testing Battery

To assess physical development, a fitness testing battery which included; lower-body power, strength, speed and cardiovascular fitness tests were conducted in line with previous studies [59]. Speed was reported at 10 and 40 m distances [60], lower-body power was reported using countermovement jump (CMJ) height (m) and strength was reported using the isometric mid-thigh pull (IMTP) [61– 63] peak force (kg) and relative peak force (kg^−1^) measures. The fitness testing battery was conducted over 2 weeks. In week 1, subjects performed measures of strength via the IMTP and power via the CMJ. In week 2, field-based measures of 10–40 m sprints were performed to measure acceleration and max velocity. On all testing days, the test causing the greatest strain on the neuromuscular system was performed first to enhance the reliability of all maximal testing procedures [64]. Completion rates: 97% for PFT1, 96% for PFT2 and 97% for PFT3.

#### Log Diary

Student athletes were asked to fill in a log diary across four timepoints in the academic year consisting of open-ended questions that explored the positive and negative holistic impacts and any features and processes of the sport-friendly school that caused, attributed or drove these impacts. Open-ended questions allowed the respondents to express opinions without being influenced by the researcher [65]. For example, student athletes were asked to reflect on the last month and outline the positive and negative impacts they had experienced on their athletic/physical, academic, psychosocial and psychological development. Furthermore, open-ended questions allowed respondents to include more contextual information, giving more feedback on the features and processes of the sport-friendly school programme that drove/facilitated positive student athlete holistic impacts [31]. For example, student athletes were asked to outline what caused, attributed or drove these impacts/outcomes to happen (e.g. what characteristics, features or processes?). Completion rates: 24% for L1, 42% for L2, 38% for L3 and 38% for L4.

#### Observational Field Notes

To achieve contextual sensitivity, emphasis was placed on participant observation of the daily lives of the student athletes in their natural setting as an essential method of data collection [66]. Over the 33-week academic term, the primary researcher completed observational field notes throughout each academic day relating to objective observations and conversations and subjective reflections of the actions, behaviours and interactions observed at ‘Nunwick High’ [67, 68]. Observations were made from a holistic viewpoint, generally attuned to the broader context of the school, including context-individual interactions and processes between sport school features and holistic athlete impacts. Notes were also taken on specific coaching actions and behaviours, individual participant experiences and the interactions observed between student athletes, coaches and teachers. The observations enhanced the researcher’s understanding of the ‘Nunwick High’ context and student athletes’ holistic development [68].

#### Timeline Diagram/Illustration

At the end of the academic year, a convenience sample of 15 participants (mixture of sport and sex) were chosen to complete a timeline diagram/illustration visualising and displaying their personal experiences of the fluctuations in academic stress and sport workload across the academic year. Within the group, each individual was asked to draw a graph representing their academic stress and sport workload across different periods of the academic year (term 1 to term 6). In addition, they were asked to highlight the key academic assessment periods across this time period. After the student athletes completed their timeline, they described and discussed their diagrams as a group, providing personal explanations and rationale for the timelines they had drawn with the primary researcher who wrote down additional notes. Successively, findings (from both quantitative and qualitative data) were fed back to participants and an opportunity was given for participants to elaborate and provide more contextual information on the findings. The data were then integrated as part of the results, complementing and enriching the data generated in the TA [69]. Although the researcher made sure to keep the discussion on topic, as well as reiterate that there were no right or wrong viewpoints [70], the direction of the discussion was driven by the student athletes. This form of research has been used in previous studies [e.g., 71] and provided student athletes with a sense of engagement and ownership over the research process.

### Data Analysis

#### Aims 1 and 2 Data Analysis

##### Quantitative Analysis

To address research aims 1 and 2, two generalised mixed models were conducted using R (Version 4.1.3). The first model (addressing aim 1) assessed the changes in impacts across the school year (33 weeks). The change in score of each holistic variable was used as the dependent variable, with time (i.e. Q1–Q5, PFT1–PFT3 and A1–A3) added as the fixed factor. Individual participants and sport were used as covariates (random factors). The second model (addressing aim 2) considered the specificity of athlete characteristics. Each holistic variable was used as the dependent variable, with biological sex (female versus male), living status (border versus non-boarder) and external sport commitment (a student athlete who played sport externally to the sport-friendly school versus a student athlete who only played sport for the sport-friendly school) added as fixed factors. Individual participants and sport were again used as covariates (random factors). The *p*-value was set at 0.05. Injury and illness incidence rates were processed separately using Excel (Microsoft Office 2021) and described using percentages with frequencies due to being bi-nominal data.

##### Qualitative Analysis

Alongside the quantitative data, qualitative data were used to evaluate the impacts of sport-friendly school involvement. The data was coded using a largely deductive approach [72]. First, during the preparation phase, qualitative data was organised and managed into categories to be analysed together (i.e. log diaries, open-ended questionnaires and observation field notes and timeline diagram/illustration transcripts) and the primary researcher obtained a sense of the whole data through reading the transcripts several times. Next, during the organisational phase, data were generated through coding [73]. Our coding approach was deductive in nature as most codes were generated through the available systematic review [8] and the online questionnaire items (refer to Table 1). Inductive coding was used as new themes specific to the holistic impacts of student athletes and any specificity of athlete characteristics were identified during the coding process.

##### Triangulation

Given that quantitative and qualitative methods were used to investigate the same holistic student athlete impacts, the data for analysis were compatible for integration using the process of triangulation resulting in the creation of a number of themes [74, 75]. As part of this process, the primary researcher compared the findings from the quantitative and qualitative analysis and considered where the findings from each method agree (converge), offer complementary information on the same issue (complementarity) or appear to contrast each other (discrepancy or dissonance) [75]. Subsequently, the assessment of convergency, complementary and discrepancy were discussed among the authors to (1) clarify interpretations of the findings and (2) determine the degree of agreement among researchers on triangulated findings [75]. Finally, after refining the themes, the primary researcher defined and named the themes.

#### Aim 3 Data Analysis

Aim 3 aimed to provide a more explanatory (i.e. seeking to explain the causes of phenomena) approach to research [76]. As such, Fryers’ [77] five-step CR approach to thematic analysis (TA) was used to analyse the qualitative data (i.e. log diaries, open-ended questionnaires, observation field notes and timeline diagram/illustration transcripts). As part of the first stage of TA, the primary author clearly outlined and refined the research aim and objective (i.e. explore the features and processes of the sport-friendly school programme that drive/facilitate positive and negative holistic impacts). In the second stage, the primary author immersed herself in the data by reading and re-reading texts to familiarise themselves with the findings and make notes on the initial thoughts and questions. Following familiarisation, stage three consisted of applying, developing and reviewing codes (step 3 [77]). Descriptive codes were applied to segments of qualitative text that were considered relevant to the research aims (e.g. features and processes of ‘Nunwick High’). Following the development of codes, step 4 entailed grouping all codes into themes [77]. Explanations were developed to suggest how particular features and processes of ‘Nunwick High’ produce the holistic impacts evidenced in the data (i.e., aims 1). Finally, within stage five [77], reflections on the overall analysis were discussed and reviewed among the research team, with a particular focus on checking the plausibility of the explanations against pre-existing evidence (i.e. in the data as well as existing theory).

### Establishing Research Rigour

Following recent recommendations, Hirose and Creswell’s [78] six core quality criteria for mixed methods studies are proposed as useful in judging the rigour of the current study. First, the authors have outlined a clear rationale for the use and appropriateness of mixed methods methodology in this study (i.e. criteria 1). Second, throughout the design included specific quantitative (e.g. What are the impacts of sport school involvement on the physical development of athletes?), qualitative (e.g. Can you tell us about the balance between sport and school?) and mixed methods (e.g. How were changes in personal development brought about by the environment?) questions (i.e. criteria 2). Third, it has been clearly outlined which elements of data collection resulted in quantitative and qualitative data, as well as how each type of data was analysed. Furthermore, quantitative data are clearly presented in Table 3, and qualitative data have been represented in direct quotes throughout the results (i.e. criteria 3). The mixed methods research design has been identified along with a diagram of data-collection moments (i.e. criterion 4). Fifth, the authors have clearly outlined how data-integration has taken place, this is then evidenced throughout the results and Fig. 3 captures a display of how findings have been integrated (i.e. criterion 5). The integration of data resulted in added value, as it allowed the authors to highlight similarities and differences between quantitative and qualitative findings throughout the results, providing a more nuanced understanding of the holistic impact of sport school involvement. Furthermore, the notion of meta-inferences (i.e. inferences that draw on both quantitative, qualitative and transcend both databases or what does it all mean together), fit very well with the CR stance of the study and the analytical process employed to formulate initial theories (i.e. explanations) as to how things worked within this sport school context (i.e. criterion 6). Finally, further in line with the CR philosophical underpinnings and aims [79], we also invite the reader to judge the findings presented in terms of their plausibility (i.e. do the offered explanations make sense, both in light of the presented data and the existing research literature) and utility (i.e. how well the research account offers predictions for likely outcomes and can be used to guide practical actions in the real world).

**Table 3:** Multidimensional student-athlete impacts over an academic year and differences by sex, boarding status and external sport commitments

		Time 1 (Intercept)^a^	Time 2^b^	Time 3^c^	Time 4^d^	Time 5^e^	Female versus Male	Boarder versus non-Boarder	Internal only versus external sport
		Mean (95% CI)	Mean (95% CI)	Mean (95% CI)	Mean (95% CI)	Mean (95% CI)	Mean Difference	Mean Difference	Mean Difference
Sport and academic	Training (h/week)	9.92^c,d,e^ (8.70–11.15)	10.23^c,d,e^ (8.24–12.23)	8.84^d,e^ (6.85–10.83)	2.22 (0.23–4.21)	1.57 (0.00–3.54)	− 1.43	− 1.10	− 2.30***
	Competitions (number/week)	2.06^d,e^ (1.57–2.54)	2.26^c,d,e^ (1.43–3.09)	1.86^d,e^ (1.02–2.68)	1.14^e^ (0.31–1.97)	0.49 (0.00–1.31)	− 1.27**	0.22	− 1.42***
	Rest days (number/week)	1.31^e^ (1.04–1.56)	1.35^e^ (0.84–1.83)	1.33^e^ (0.83–1.81)	1.42^e^ (0.92–1.90)	2.00 (1.5–2.47)	0.07	0.07	0.52**
	Academic Hours (h/week)	28.1^c,d^ (25.9–30.4)	26.7^c^ (21.5–31.8)	23.6^e^ (18.5–28.7)	25.2^e^ (20.10–30.4)	28.2 (23.2–33.3)	4.48	− 0.37	− 0.80
	Lessons missed (number/week)	0.57^b,c,d^ (0.00–1.17)	2.01^e^ (0.68–3.34)	2.48^e^ (1.16–3.81)	2.51^e^ (1.18–3.84)	0.77 (0.00–2.08)	− 0.11	0.11	-0.60
	Difficulty balancing academic and sport workload	2.90^b,c,d,e^ (2.68–3.11)	3.34^e^ (2.85–3.82)	3.20^e^ (2.72–3.68)	3.22^e^ (2.73–3.69)	2.43 (1.95–2.90)	− 0.22	0.23	0.38
	Academic motivation	4.82 (4.58–5.08)	4.87 (4.45–5.31)	4.88 (4.47–5.32)	4.85 (4.43–5.29)	4.78 (4.36–5.21)	0.36	0.19	0.21
	Student athletic motivation	4.58^d,e^ (4.23–4.94)	4.44^e^ (3.92–4.98)	4.43^e^ (3.91–4.96)	4.31 (3.79–4.84)	4.20 (3.68–4.72)	− 0.37	0.00	0.10
	Career athletic motivation	2.62 (2.38–2.88)	2.66 (2.21–3.14)	2.79 (2.33–3.25)	2.75 (2.30–3.22)	2.68 (2.23–3.15)	− 0.04	− 0.03	− 0.30
Academic	Academic assessment grades	4.29^e^ (4.11–4.47)	4.44 (4.08–4.79)	4.46 (4.11–4.81)	–	4.57 (4.22–4.92)	− 0.05	0.13	0.16
Athletic/physical	Sport competence	3.59^c,e^ (3.45–3.73)	3.58^c^ (3.31–3.86)	3.40 (3.13–3.68)	3.48 (3.21–3.76)	3.44 (3.17–3.72)	− 0.39*	0.01	0.03
	Sport confidence	2.89 (2.70–3.09)	2.85 (2.51–3.18)	2.93 (2.60–3.26)	2.84 (2.51–3.17)	2.88 (2.56–3.21)	0.26	0.14	− 0.03
	General stress	2.14 (1.72–2.55)	2.22 (1.62–2.80)	2.28 (1.69–2.87)	2.21 (1.61–2.79)	2.15 (1.55–2.73)	1.03***	− 0.21	0.15
	General recovery	3.37 (3.19–3.56)	3.42 (3.07–3.76)	3.32^e^ (2.98–3.66)	3.36 (3.02–3.71)	3.49 (3.15–3.83)	− 0.47*	0.02	− 0.32*
	Sport-specific stress	2.03^c^ (1.84–2.21)	2.14^e^ (1.77–2.51)	2.22^e^ (1.85–2.58)	2.14^e^ (1.77–2.50)	1.94 (1.58–2.30)	− 0.08	− 0.07	− 0.02
	Sport-specific recovery	3.20^c^ (3.00–3.39)	3.17 (2.80–3.54)	3.02 (2.65–3.38)	3.11 (2.73–3.47)	3.04 (2.67–3.40)	− 0.31	0.07	− 0.09
	Injury incidence	32%	47%	39%	37%	23%	–	–	–
	Illness incidence	31%	20%	23%	23%	11%	–	–	–
	10 m (s)	1.89 (1.77–2.02)	1.99 (1.75–2.23)	–	1.93 (1.70–2.16)	–	0.24	− 0.01	0.02
	40 m (s)	5.87^b^ (5.53–6.20)	5.82^d^ (5.43–6.20)	–	5.89 (5.51–6.26)	–	0.93***	− 0.02	+ 0.02
	CMJ (cm)	34.3^b,d^ (30.0–38.6)	36.1 (31.0–41.3)	–	36.5 (31.3–41.6)	–	− 10.49***	− 0.88	0.88
	IMTP (kg)	123.3^b,d^ (97.3–149.0)	130.4^d^ (99.8–160.7)	–	160.6 (130.0–191.0)	–	− 50.96***	− 6.41	0.32
Psychosocial and psychological	Life skills	–	3.51 (3.36–3.67)	3.51 (3.27–3.76)	3.59 (3.35–3.84)	3.56 (3.33–3.82)	0.28	− 0.11	− 0.04
	Resilience	3.36 (3.22–3.51)	3.35 (3.10–3.61)	3.29 (3.04–3.54)	3.34 (3.09–3.60)	3.39 (3.15–3.65)	− 0.23	0.09	0.10
	Social support and peers	14.9 (12.2–15.8)	15.5 (14.1–17.0)	15.4 (13.9–16.8)	15.0 (13.5–16.5)	15.1 (13.6–16.5)	− 0.43	1.32	0.05
	Family and free time	17.6 (16.7–18.5)	17.6 (15.9–19.2)	17.5 (16.4–19.7)	18.1 (16.4–19.7)	17.9 (16.3–19.6)	− 1.05	− 0.75	− 0.83
	Body image	7.87 (4.10–11.54)	6.88 (1.82–11.83)	7.58 (2.56–12.51)	7.49 (2.45–12.44)	6.76 (1.75–11.67)	9.28***	− 2.43	0.56

AM, academic motivation; SAM; student athletic motivation, CAM; career athletic motivation; CMJ, countermovement jump; IMTP, isometric midthigh pull

^a^Significantly different to time 1

^b^Significantly different to time 2

^c^Significantly different to time 3

^d^Significantly different to time 4

^e^Significantly different to time 5

***P < 0.001

**P = 0.001

*P = 0.01

## Results

In line with the study’s aims, the results are presented in three higher-order themes: (3.1) longitudinal investigation of student-athlete holistic impacts, (3.2) specificity of athlete characteristics and (3.3) features and processes of the sport-friendly school program (i.e. what worked for whom and how).

### Longitudinal Investigation of Student-Athlete Holistic Impacts

The triangulated holistic student-athlete impacts are presented below. Table 3 presents the quantitative statistical results for each impact at each timepoint. Furthermore, differences in student-athlete characteristics (i.e. sex, boarding and external sport) are presented. The descriptions below triangulate the quantitative and qualitative data within key themes to present the longitudinal holistic impacts.

#### Fluctuations in Academic and Sports Workload Over-time Culminate in a Variety of Impacts

Table 3 presents how sports training, competition frequency and the number of rest days changed across the academic year. Sport training and competition frequency significantly decreased in March and May (1.57–2.22 h/week and 0.49–1.14 competitions/week) compared with September–February (8.84–10.23 h/week and 1.86–2.26 competitions/week). Significantly more rest days were experienced during May (~ 2.00 per week) than in the other periods. This finding is supported by the student athletes’ timeline diagrams/illustrations whereby most student athletes’ sport workload was typically high across terms 1–4, with a drop off in terms 5 and 6. In contrast, for summer sports such as cricket and athletics, the highest sport workload appeared in term 6 when they were also doing their final academic examinations, as exemplified by a summer sport student athlete when talking about term 6: “I think for [summer sport] it is hard. We literally will have three games a week and two exams a week”.

Fluctuating patterns were also shown for academic hours (represented by hours spent in academic lessons plus hours doing home work) and number of lessons missed. Academic hours were significantly lower during November/December and February (23.6–26.7 h/week) and highest in September and May (~ 28 h/week), which coincided with the number of lessons missed (i.e. more lessons missed in November–February than September–May). Furthermore, when the student athletes were describing their timelines, they highlighted three time periods that could be considered the most stressful from an academic perspective: (1) the second week back after the Christmas break (mock exam week), (2) the final 2 weeks before Easter (final coursework deadlines) and (3) the whole of terms 5 and 6 (final academic examinations).

##### Periods of Difficulty Balancing Dual Demands and Changes in Stress and Recovery

Student athletes found balancing academic and sports workload significantly harder during November–March (3.22–3.34) and easiest during May (2.43). When student athletes were describing their timelines, they described a constant oscillation between periods of high academic stress (e.g. assessment time, mocks and exams) and high sport workload (e.g. busy fixture list, major tournaments and finals), with them often coinciding, resulting in increased stress and pressure.

“So, at the moment it is fine, but now gradually, academics are getting a lot more pressure on and the fixtures start to go like that again [demonstrated a steep incline with hand]. And then there is not really a break till March and by then should be absolutely ready for your A-levels and you are behind. Still revising some topics”.

Although student athletes’ general stress stayed stable across the academic year (no significant change across September to May), sport-specific stress levels varied across different time periods (highest in February and lowest in May). Regarding recovery, although general recovery stayed relatively stable across the academic year, sport-specific recovery was significantly lower in February compared with September (implying that student athletes were not recovering as well from sports during February compared with September).

##### Fatigue Accumulation, Culminating in Student-Athletic De-motivation

At the beginning of the academic year (September), student athletes were exposed to an immediate high academic and physical workload (i.e. 9.93 training hours/week and 28.1 academic hours/week). Additionally, from a physical fitness perspective, student athletes are physically less fit. Overall, the initial challenges (i.e. demanding schedule) and lack of physical fitness appeared to result in student athletes feeling fatigued, both mentally and physically at the start of the academic year. For example, a student athlete stated in their log diary 3 weeks into term 1:

“I’m keeping up with my school work but the workload is high due to not having free periods (because I play sport). I feel motivated to improve in both my academics and my sport. I am finding myself feeling more tired during the week but this is probably a combination of higher amounts of physical activity and not going to bed early enough.”

The feeling of fatigue was a common impact across the academic year. The student athletes frequently stated in their log diary that they were ‘always tired’, as exemplified by this student athlete: “I always want to sleep”. This impact was further exaggerated for student athletes with increased academic demands (e.g. undertaking four A-levels versus three), as exemplified by one student athlete’s log diary:

“The workload is high because I am taking 4 A-levels as well as doing my sport throughout the day- this means I have less time in school to complete work set and have to do the majority of it at home. This can build up and occasionally I find myself working until late which is leaving me feeling tired in the morning”.

Finally, there seemed to be an accumulative build-up of fatigue towards the end of each academic term and year. From a conversation with one of the coaches at the end of term 2, they stated: “This time of year everything changes. Kids getting tired, we are getting tired and boredom setting in”. The effect of fatigue on student athletes’ academic work was further elaborated on in a conversation with a student athlete: “I think there is enough time to do your work, it is just not enough time where you are not tired. You come home and you are knackered you don’t want to do work.” The student athletes described becoming demotivated during the end of term with a lack of physical development. “I plateaued. I started hating [sport]. I wasn’t improving, I was tired, I was stressed. To the point where I didn’t enjoy it”.

The feelings of de-motivation and mental and physical fatigue were further exaggerated in terms 5 and 6. A student athlete stated: “It is a bit burnout. You go, boom, boom, boom, boom, boom and now you just feel like flat”. By terms 5 and 6, student athletes appeared to have a lack of motivation and burnout for performance sports (consistent with student athletic motivation score, which was significantly lower in May), where student athletes wanted a period of unstructured training and time away from the performance environment.

“The last summer term with exams. I remember that first weekend after school finished, I literally couldn’t do anything else. I was so tired, like mentally and physically. And then I dunno, the feeling was awful”.

##### Immediate and Multiple Stresses

New student athletes at ‘Nunwick High’ experienced increased stress and pressure from an immediate intensive level of training and increased academic demands. In addition, they reported emotional and social stress from moving away from home, family and friends into a new environment. For example, from conversations with new student athletes who had transitioned into the school, they stated that they found the workload (both academic and physical) ‘a lot more’ than previous experiences. “You’re sort of chucked straight into it and expected to do everything basically. It is quite intense and a lot asked of you. Kind of have to do it and get it done”.

Then across the academic year at ‘Nunwick High’ there was evidence of three types of stressors: (1) Competitive stressors related to the demanding game schedules. “The upcoming matches that have been occurring have caused me to become more stressed”. (2) Organisational stressors from commitments to school sport balance.

“But I think sometimes, yeah it happens, but you are not enjoying it, it doesn’t become enjoyable it just becomes stressful. To go to a match and then come back and do your work. It is then not an enjoyable period”.

Finally, (3) Personal stressors when student athletes sacrifice social life for sport.

“I’m not as social as I was at the beginning of the year, I think this is due to the stress given by school. I feel as though I need to spend more time doing school work compared to socialising”.

However, contradictory to the personal stressors, student athletes’ friends and family and free time KIDSCREEN-27 Health Questionnaire scores stayed stable across the academic year.

##### Despite Challenges and Academic Pressure, Student Athletes Generally Achieved Good Academic Grades

As highlighted above, student athletes experienced challenges across the academic year (e.g. demanding schedule, fatigue and multiple stressors), in addition to academic pressure, as highlighted by a student athlete, “for me, academic pressure is a really big thing, because I am really scared, I am going to let it slip accidently”. Despite these challenges, overall, academic grades stayed relatively stable across the academic year (4.29–4.57), with only June significantly higher than October. This finding coincides with the fact that academic motivation also stayed stable across the academic year (4.78–4.88). This is further supported by the qualitative data which highlighted that student athletes at ‘Nunwick High’ generally achieved good academic grades. According to the log diaries, although some challenges around managing the multiple demands on their time were highlighted, most student athletes were generally happy with their academic development across the year: “I think I have developed academically in my exams; I have improved consistently throughout the year”.

#### Sport Performance Development and Well-Being Across the Year

As highlighted in the previous theme (3.1.1.2) student athletes are physically less fit at the beginning of the academic year. However, over time there were significant improvements in IMTP strength (123.3 kg September and 160.6 kg in March/April), CMJ height (34.3 cm in September and 36.5 cm in March/April) and 40 m max velocity (only September–December), whilst 10 m acceleration stayed stable.

Sport confidence was stable across the academic year (no significant change from September to May). However, there was a significant decrease in student athletes’ perceived sport competence during February (3.40) compared with September–December (3.59–3.58). Sport competence then recovered between February to May but not compared with September–December levels. This data contradicts the qualitative findings whereby student athletes largely expressed how being involved in the performance sports programme had resulted in them becoming better at their sport. They stated in their log diaries that they could see improvements in their physical, technical and tactical development and overall sporting performance across the academic year.

“My athletic development has gradually improved over time during all of the training and sessions. My physical development has improved slightly as well, especially with things like speed and size. My personal fitness has improved from the training and has encouraged me to do more out of the sessions”.

When evaluating injury and illness, injury incidence was higher than illness incidence. The greatest number of injuries occurred in November/December (47%), with the lowest injury incidence in May (23%). Illness incidence was highest in September (31%) and lowest in May (11%). Finally, although there was no significant difference or change in the student athletes’ EAT-26 score across the academic year and average scores were below 10, there were student athletes who scored ≥ 10, signifying disordered eating behaviour and attitude.

#### Personal Development

Student athletes also reported to have developed personally, although LSSS (3.51–3.56) and resilience (3.29–3.39) scores stayed stable across the academic year (no significant change across September–May). Through the qualitative data many student athletes emphasised they had developed a range of life skills and attitudes they could use both within and outside of sport. For example, they felt they had become more confident and developed their communication, social integration ability, social skills, work ethic, motivation, time-management skills, teamwork and leadership skills, in addition to becoming more independent, resilient, disciplined, mature and responsible adults. Student athletes highlighted these developments in their log diaries and through conversations with the primary researcher:

Student athlete 1: “Allowed me to develop my motivational skills. Training more and work in the gym helped to develop my social skills and my physical and mental abilities of perseverance during training and during my school work”.

Student athlete 2: “We talked about balancing a lot and if you are doing sport and academics, you kind of naturally build the skill of time-management and balancing stuff. I have got sport and A-levels as well, so I kind of have to think about time management as well. So, after I finish my sport, I know I need to go home and complete my prep. So, I kind of manage my day to get it all done”.

### Specificity of Athlete Characteristics

There was no significant difference between boarders and non-boarders across all variables. For sex, females had significantly fewer weekly competitions than males. Female sport confidence scores and general recovery scores were significantly lower (− 0.68, − 0.47) and general stress and EAT-26 scores were significantly higher (+ 1.03, + 9.28) than males. Finally, females had significantly lower CMJ (− 10.49 cm) and IMTP (− 50.96 kg) and significantly slower 40 m max velocity (+ 0.93 s) scores than males. Internal sport-only student athletes had significantly less training (− 2.30 h/week) and competitions (− 1.42 number/week) and more rest days (0.52 number/week) per week compared with external sport student athletes. However, internal-only student athletes’ general recovery was significantly lower (− 0.32), which contradicts the qualitative findings where student athletes who played sports externally and for the sport-friendly school expressed feeling particularly fatigued and lacking rest and recovery.

### Features and Processes of the Sport-Friendly School Program

While the primary aim of this study is to evaluate sports school holistic impacts, the third aim is to gain insight into the context-individual interactions underpinning them (i.e. features and processes). Accordingly, this section aims to provide a narrative overview describing insights into particular features and processes of ‘Nunwick High’.

#### Importance of Personal Motivation, Value of Education and Academic Support Services

Student athletes stated that they achieved good academic grades due to developing their personal motivation, organisational skills and commitment (i.e. hard work ethic, determination, self-motivation, developing a revision routine and creating a timetable of free time to balance workload), as highlighted in a student athletes log diary: “My work ethic and motivation have improved, which has caused me to work harder and put in more effort. I am not afraid to ask questions anymore to help me understand”.

Secondly, coach support was highlighted to assist student athletes’ academic development. There appeared to be flexibility with sports training and support from the coaches around the periods of high academic stress (i.e. student athletes were allowed to miss training sessions to do work), as exemplified by a student athlete: “Since my coaches have understood about me wanting to focus on my work, sometimes it has been helpful as I know that they support me”.

Finally, the student athletes received extra academic support. Teachers and fellow pupils provided extra tutoring (i.e. one-to-one help) in their own time. Teachers provided subject and revision clinics, and ‘Nunwick High’ had a learning development department. The extra academic support provided is demonstrated in the following quote from a student athlete’s log diary:

“Getting help from teachers—one-to-one help. Clinic revision—weekly revision after school to revise through any topics that I am not comfortable with. Microsoft Teams—online teams in which I can message my teachers directly whenever I am stuck”.

#### Performance Sports Program with Direct Sport-Related Practices, Staff and Support Services

‘Nunwick High’ was reported and observed as having high-quality facilities, fixtures, coaching staff and training partners. Student athletes had access to professional, high-quality facilities (e.g. a fully equipped gym, pool, indoor three-court sports hall and numerous astroturfs and grass fields). The performance sports program arranged high-level fixtures against top opposition (e.g. academy teams, high-level clubs and top sports schools). As a result, the student athletes were challenged technically, tactically, physically and psychologically against high-level opposition, as attributed by a student athlete in their log diary: “Recent fixtures, tournaments and matches have positively impacted my development, lifting to my maximum potential and pushing myself in court sessions”. Moreover, ‘Nunwick High’ employed high-quality coaches who could provide expert coaching, support and education to enhance the sporting development of the student athletes further. ‘Nunwick High’ was also described as attracting a big pool of talented student athletes providing high-quality training partners/teammates who acted as influential mentors—providing a high-quality training and learning environment where student athletes pushed their peers to be better and develop from one another. For example, a student athlete stated in their log diary:

“My skill and physical capabilities have improved drastically over the past month as the combination of regular strength and conditioning sessions as well as daily access to an indoor basketball court and high-quality players and coaching staff has driven me to become a completely different basketball player”.

As highlighted in the qualitative and quantitative data, the student athletes trained regularly across the year. As a result, the student athletes had more opportunities to practice, play and develop in their sport. For example, a student athlete stated they had ‘developed as a player’ and that this was due to ‘training every day and having games regularly’.

‘Nunwick High’ also had a multi-disciplinary sports staff as part of the performance sports program (i.e. SC, physiotherapist and nutritionist). The student athletes had designated and regular SC sessions within their school timetable, where the SC staff provided them with tailored and sport-specific physical development programs. Additionally, the SC staff provided additional athletic and physical development resources (e.g. cardiovascular fitness sessions, advice on recovery, mobility sessions) and put on recovery sessions (e.g. stretching/yoga). This support was deemed to positively support the student athlete’s athletic and physical development. For example, a student athlete, when answering in their log diary what was the driving factor for their improved athletic and physical development, stated:

“I have had a personal SC programme fitted to what will help me make the biggest impact on my sport; this has been essential for me and helped me to push hard, knowing that my interests are being taken care of and frequently adapted to fit my needs and any progress that I make”.

Whilst another student athlete stated the support available when injured:

“Due to an injury, I haven’t been able to train as often as normal on the pitch; however, the programme has still been able to help me develop during this time. I have had a lot of physio sessions which have helped me understand what is wrong with me, and the physio works closely with the SC staff, who are then able to provide me with stretches related to my injury as well as exercises that help my performance whilst taking into consideration my injury/limitations”.

#### Because the Environment Demanded It

The requirement to take accountability and responsibility, live away from home, and the busy schedule of sports and academics required student athletes to manage themselves effectively, become better at managing multiple demands and be disciplined.

“Time-management as well. You don’t necessarily get taught it. But you learn it by having such a busy schedule. You have to work out what to do when”.

The school strongly focused on giving the players accountability and responsibility for their academic and sport development. As described above, an environment was witnessed where the student athletes were given the relevant tools to help aid their sporting development and academic development. The student athletes were responsible for using these resources and maximising the opportunities in their own time.

“Environment where everything the athletes need is available to them (e.g. video from games, SC, yoga, extra sessions, academic support, pastoral care), but although the athletes are encouraged to utilise everything that is on offer to them, it is the athlete’s responsibility on how they use their time and if they utilise their time here effectively”. [Field note, 03/02/2022]

However, there was a lack of upskilling to allow student athletes to maximise their development, particularly in managing their time effectively. The student athletes felt that sometimes, the staff presumed they had the relevant skills without providing them with the tools to facilitate appropriate ownership of their development (i.e. feeling left to their own devices). For example, student athletes stated the following comments when talking about taking responsibility:

Student athlete 1: “I think they just expect you to be more organised, to be able to fit your sport in”.

Student athlete 2: “What we get offered here, most of us haven't been exposed to it before coming here. Then you are expected to know how to use it. When a lot of people don’t. So, then they don’t get the most out of as they can do”.

Furthermore, the additional work student athletes were expected to do in their own time (e.g. clip their own video) adds to their workload, providing further conflicts with their academic study and personal time.

“Yeah, like no one tells you to go and watch the video. But I like to watch it and see what happens and see why we lost to [team]. But then that is an hour, hour and a half of Thursday when the video comes out. So that is when I should be working”.

#### Lack of Organisation and Planning of Training Load

When the student athletes first joined the performance sport programme in the sixth form, they transitioned into an intensive level of training. There was no preseason at the sport-friendly school, so the student athletes were immediately exposed to a high physical workload. Furthermore, first-team fixtures were organised within the second week of the term. When asked if the student athletes liked having fixtures within the second week of term, there was an overwhelming ‘no’ feeling. Student athletes stated they were ‘not adequately prepared’ and ‘had not had enough training time together’.

Additionally, from a physical fitness perspective, when student athletes transitioned into ‘Nunwick High’, or returned at the beginning of the academic year, they felt physically ill prepared for the immediate, intense training load. Through pre-season physical fitness testing, the primary researcher observed student athletes coming back from the summer holidays with lower physical fitness levels than expected.

“Just completed 30–15 running fitness test with [sport]. Generally, the student athlete’s cardiovascular fitness scores are lower than I would expect them to be at the beginning of the year in comparison to normative, expected data for their sport”. [Field note, 07/09/2021]

Coaches, in conversation with the researcher, emphasised that at the beginning of the year ‘students were not fit enough’. A student athlete further highlighted this comment when talking about the initial start of term: “And also, our fitness isn’t as good as it would have been after training all the time at school. So, I think we are lot more unfit as a lot of us don’t train outside of school”.

Within an academic year at ‘Nunwick High’, no periodised planning, tapering or deload was scheduled within the performance sport programme across a term. The primary researcher observed a lack of balance between high training loads followed by intentional low training loads (i.e. deload/tapering weeks).

“Season at [Nunwick High] is very full on and intense the whole time. There is no periodised planning, tapering or deload week within the term or season. This is resulting in kids being exhausted by the last 2 weeks”. [Field note, 15/03/2022]

“Sometimes, I think we over train. Like having 2 h on Wednesday, then another 2 h on Thursday. Then an hour on Monday, SC on Tuesday and another SC on Friday. With no recovery. You know, it is really intense”.

Despite the benefit of offering high-quality competition from a sporting development point of view, ‘Nunwick High’ appeared to enter every competition, league and cup and has an extensive list of friendly competitions. As a result, some sports teams had two (on the rare occasion, three) internal sports fixtures a week (not considering the fixtures some student athletes have externally outside of school), leading to potential fixture congestion. Based on observations and log diaries, the extensive fixture list appeared to put further pressure on student athletes academically, as they missed many lessons and were fatigued. As exemplified by a student athlete in their log diary, “Having regular away fixtures has caused me to miss multiple lessons every week and afterschool training has limited time to catch up on homework”.

Finally, the primary researcher observed a lack of collaboration with external sports schedules. For example, based on her observations, the primary researcher reflected:

“Given that match play required a longer period of recovery than training, the school coach on Thursday often incorporated recovery sessions. However, unaware of the school match the previous day, one student athlete’s club team continued with an unmodified training session, including one to two hours of technical training on a Thursday night. As a result, negating the benefits of the recovery sessions within the school. The student athlete returned to school training on Friday, 24 h after the match, with the school coach presuming the fatigue from the match had largely dissipated. From chatting to the student athlete, they stated that they did not actually have the chance to recover from the match on Wednesday and entered the weekend fixtures feeling fatigued, which he believed compromised his performance”. [Field Note, 17/02/2022]

#### Lack of Coordination and Program Flexibility Between Academic and Sports Timetables

Although academic support services were available and some academic staff provided extra academic support and understanding for the student athletes when needed, there appeared to be a need for more understanding from all teachers. For example, in a conversation with two athletes, they stated:

Student athlete 1: “If you miss a lesson, then they just send you the work and expect you to do it yourself”.

Student athlete 2: “Sometimes I don't think my academic teachers understand. They are like ‘again, really’. I am like; it doesn’t change just because I did it last week”.

**Fig. 2:**
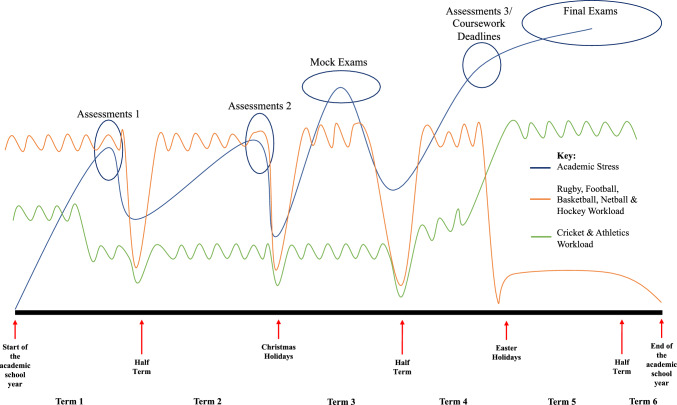
Overview of oscillations in academic stress and sport workload across the school year

Moreover, although there appeared to be flexibility with physical training and support from the coaches around periods of high academic stress, there was a lack of planning, co-operation and compromise with scheduling, with sports fixtures clashing with periods of high academic stress (apart from in term 6). For example, as highlighted in Sect. 3.1.1 student athletes described a constant oscillation between periods of high academic stress and periods of high sport workload with them often coinciding, resulting in increased stress and pressure (as depicted in Fig. 2 which summarises the general patterns observed in the student athletes’ timeline diagrams/illustrations).

“I think my timetable doesn’t match up. So that means I get assessments and work I miss because I go to matches, and then I am still going to the gym and stuff like that. So, it feels like there is no compromise, and when it comes to assessments, I still feel like I need to do the match”.

Despite academic flexibility/support by some coaches and teachers, due to the conflicts between academic and sport schedules, student athletes often felt conflicted, pressured and guilty towards both coaches and teachers if they chose one endeavour more than the other and were often reminded of it. “[Coach] will mention past things you have done. Like, yeah, but you didn't come to this one either, and you didn't come to this one, and now you are missing this one. So yeah, like the build-up of guilt”. Additionally, there seemed to be a lack of understanding and a conflict between what is a priority for student athletes regarding internal and external training, with student athletes feeling scared to come forward if they were tired.

“I feel like if I said to [coach], I can’t train as well on Wednesday as I was training for [club] on Tuesday. Then he would like to quit [club]. And I don’t want to quit it. But I don’t think I could come forward and say I was tired because I trained last night. As he would say that I am disrupting [school sport]. You are here to play [school sport]”.

There was little evidence of direct communication and alignment between sport coaches and teachers, where they worked together to ensure that their schedules were appropriately adjusted and aligned to the student’s academic (deadlines and submissions) and sport (tournaments and cup competitions) load. Instead, student athletes explained that they were the ‘middle ground’ for communication between coaches and teachers.

“I think the only thing that is hard about it is communication between your teachers and the coaches as well. As obviously, the teachers will have their say and be like, ‘You do too many matches’”.

Finally, student athletes at ‘Nunwick High’ had varying academic demands, extra-curricular activities and sporting commitments. Moreover, the performance sport teams’ schedules varied weekly (e.g. a team may compete in three competitions 1 week and no competitions the following week). Despite this, there appeared to be an overall ‘one size fits all’ approach to the overall planning, with a lack of adaptation to individual student athletes’ varying commitments and between-week team schedules, causing further competing demands and stress.

“Yeah. I think when we had gym and dance. That was sort of like a commitment. [Coach] would know we would have it after school but still expect me to go 100%, even though the night before we would have had a full run-through and everything went wrong and duh duh duh, school production itself. So it is sort of, I understand you have all this other stuff, but it doesn’t give you an excuse not to go 100% in training”.

## Discussion

To our knowledge, this study is the first to longitudinally evaluate (1) the impact of sport-friendly school involvement on the holistic development (i.e. academic, athletic, psychosocial and psychological) of student athletes, (2) the holistic impact according to the specificity of student athlete characteristics (i.e. sex, boarding status and external sport involvement) and (3) the features and processes of the sport-friendly school programme that drive/facilitate positive and negative holistic impacts.

**Fig. 3:**
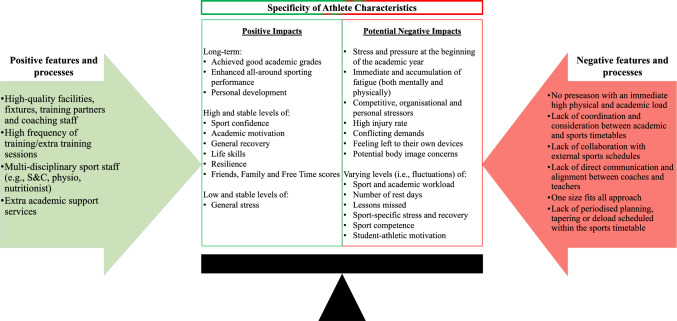
Summary of the longitudinal holistic impacts of sport-friendly school involvement and the potential features and processes that drive/facilitate positive and negative holistic impacts

Overall, mixed-method data demonstrated that over-time student athletes, achieved good academic grades, enhanced their all-round sporting performance and developed personally, demonstrating positive short-term and potential long-term positive impacts of sport-friendly school involvement. In addition, student athletes’ sport confidence, academic motivation, academic grades, general recovery, life skills, resilience and friends, family and free time scores remained stable and relatively high across the academic year. Potential features and processes of ‘Nunwick High’ that contributed to these positive impacts included: high-quality facilities, fixtures, training partners and coaching staff, high frequency and extra training, multi-disciplinary sport support staff (e.g. SC, physiotherapist and nutritionist), academic support services, and self-reported motivation and hard work ethic to engage with training and academics. Despite these positive benefits, the simultaneous pursuit of academic and athletic achievements provided challenges for student athletes across an academic year. Potential negative impacts found included: increased stress and pressure at the beginning of the academic year, immediate accumulation of fatigue (both mentally and physically), competitive, organisational and personal stressors, high injury rates, potential body image concerns, conflicting demands and feeling “left to their own devices”. Furthermore, student athletes’ experienced significant fluctuations in their sport and academic workload, rest, academic lessons missed, sport-specific stress and recovery, sport competence and student-athletic motivation scores across the academic year. Many of the potential challenges/negative impacts student athletes experienced seemed to be attributed to a lack of (1) gradual increase in training exposure (intensity, frequency and volume) at the beginning of the academic year, (2) coordination and consideration between academic and sport timetables, (3) collaboration with external sport schedules, (4) direct communication and alignment between the coaches and teachers, (5) program flexibility and (6) periodised planning, tapering or deload scheduled within the sport timetable. However, it is worth noting that individual characteristics shaped the sport school experience and its impact on the holistic development of student athletes. Biological sex and external sport commitments were shown to influence student-athlete holistic impacts, however boarding status did not. Figure 3 summarises the longitudinal holistic impacts of sport-friendly school involvement, including the program’s features/processes driving positive and negative impacts.

### Longitudinal Investigation of Student Athlete Holistic Impacts

#### Immediate and Intermediate Risk and Challenges

The student athletes faced numerous challenges at the onset of the academic year (e.g. high physical training loads, frequent sport fixtures and psychosocial adjustments) aligned to existing research [8]. Longitudinal data suggested these continued throughout the academic year. The workload challenges are similar to previous research in sport schools [80–82] and youth sport [12, 83] but providing sport and academic load simultaneously emphasises the challenge of combining student athletes workload with external sporting commitments. These workload challenges potentially contribute to various other impacts experienced by student athletes, such as increased rates of missed academic lessons, heightened susceptibility to injuries, and the ongoing struggle to effectively balance their athletic commitments with academic responsibilities. Consequently, this confluence of demands often results in elevated levels of fatigue, persistent feelings of tiredness, and heightened stress among student athletes. This explanation is plausible given previous literature (e.g., [84] and [85]) has emphasised that the time commitments associated with combining education alongside sports training were a crucial contributor to fatigue accumulation and stress.

The longitudinal data highlights, student athletes’ need to negotiate many fluctuating academic and sport demands and expectations across a school year, which are often conflicting [84, 86, 87]. In parallel, student athletes seemed to find the sport–academic balance easier when they had increased rest and reduced training/competitions. These findings are unsurprising, as fewer competing demands exist. Previous research similarly demonstrates that the commitment (i.e. time and effort) to sport coincide with youth athletes’ education [21] and competitions/training, resulting in youth athletes missing school for several days or even weeks/months a year [88], making balancing both sport and education challenging [9–11].

Finally, the qualitative data reveal a consistent cycle between periods of high academic stress, such as assessment times and exams, and periods of intense sports workload, such as busy fixture lists and major tournaments. These overlapping demands potentially contribute to three main categories of stress: competitive stress due to game schedules, organizational stress from balancing school and sports, and personal stress involving social sacrifices. This pattern is supported by the correlation between changes in student athletes’ training loads and their sport-specific stress levels throughout the academic year. Competitive, organisation and personal stressors are supported by Kristiansen and Stensrud’s [85] study, which found evidence of all three stressors among youth female handball sport school athletes.

#### Long-Term Positive Impact

This study suggests that despite the challenges (e.g. balancing both sporting and academic commitments) student athletes within sport schools can excel in both sport and academics. Student athletes maintained stable and high academic grades throughout the year, supported by the qualitative data. These findings are congruent with broader youth sport research, which has indicated that student athletes excel in education (e.g., [89]). However, these findings contradict previous sports school literature [81, 90], which suggested that sport participation negatively affected student athletes’ academic success.

Regarding athletic impacts, physical fitness data also demonstrated enhanced strength, speed and power. These results align with Beckmann et al. [91] study, showing increased fitness measures in student athletes enrolled in a sport school over 5 years. While the student athletes’ sport competence scores dipped compared with baseline throughout the academic year, qualitative findings demonstrated that student athletes felt they became better athletes (technical, tactically and physically). These findings may be explained by student athletes perceiving themselves as getting better but also had enhanced (different) perceptions and judgment as to where their own skills lay in comparison to others. Over time, student athletes may enhance their capacity for self-reflection and the evaluation of their abilities in comparison to others (i.e. their self-evaluation becomes increasingly more accurate but also more negative [92]), which could influence the self-perceived ratings of their own sport competence.

Finally, although student athletes’ psychosocial scores did not improve across the school year, they were relatively high at baseline and remained stable. Qualitative data highlighted the development of life skills and attitudes applicable in and beyond sport, reinforcing this trend. Previous sport school literature [82, 93, 94] supports the idea that sport school involvement fosters qualities and skills applicable to various aspects of life. Furthermore, overall LSSS scores were similar to that of British youth sport [95] and sport high school [96] student athletes. As such, sport-friendly schools should continue to develop student athletes technical, tactical, physical and academic capabilities but additionally develop their personal, social and life skill capabilities [97], to ensure student athletes develop transferable skills for life beyond the sport-friendly school environment [98].

### Specificity of Athlete Characteristics

Sex and external sport commitments were shown to influence student athlete holistic impacts, however boarding status did not. In accordance with O’Connor et al. [99], females demonstrated lower levels of sport confidence and perceived competence compared with males, along with higher general stress, lower general recovery and greater body image concerns. Literature suggests that youth athletes, particularly females, are becoming concerned about their body image at increasingly early ages [100] and body-related shame and guilt are increasing over time among female youth athletes [101]. Looking at the inter-relationship between variables, previous research has found a significant relationship between body image and sport-related variables (e.g. sport confidence [102]). Furthermore, Murray et al.’s [103] study found a significant association between higher body dissatisfaction and higher ratings of peer stress and lower self-esteem. Given the potential heightened vulnerability in females, further research should explore the holistic development of female student athletes in sport schools.

Student athletes (such as those at ‘Nunwick High’) often participate in multiple sport or for various teams within the same sport [33, 104]. External sport involvement increased student athletes’ time commitments (more training hours and competitions and less rest), intensifying the competing demands between academic and athletic pursuits. The additional demands link with lower general recovery scores for external sport student athletes. Research demonstrates that student athletes with higher weekly training loads have higher recovery-stress states than student athletes with lower weekly loads [105]. Furthermore, the qualitative data highlighted further fatigue and recovery challenges amongst this group, exacerbated by unsynchronized schedules between external and internal sport commitments. Previous research supports this conclusion, which demonstrates the ‘tug of war’ scenario of various weekly sport commitments, which can result from separate and contrasting athlete-focused training plans and goals [33, 104]. Collaborative management of training schedules among the various stakeholders (i.e. coaches) is crucial to prevent fatigue, overreaching and injury risks among this specific group [106–109], requiring aligned training aims, load management, fixture lists and flexible programming [33].

### Features and Processes of the Sport-Friendly School Program

As DC environments are complex and dynamic, whereby student athletes have to interact with many features and processes of a sport school, this study aims to advance on existing research to understand what facilitated and drove the positive and negative impacts. This approach was a unique and novel aspect of this study resulting in five key findings as discussed below.

#### Importance of Personal Motivation, Value of Education and Academic Support Services

One clear positive impact was that student athletes’ academic performance was high and stable consistent with previous research [89, 110]. These findings may be explained by the student athletes displaying stable and relatively high levels of academic motivation across the school year and personal attributes aligned to academic work (e.g. hard work, organisation skills and commitment). Research (e.g. [111]) supports the associations between individual traits (e.g. AM, educational goals and commitment) and academic achievement demonstrating that student athletes’ academic motivation is important to achieving academic success. Furthermore, academic performance may reflect the importance of the additional support offered by sport schools (e.g. extra tutoring, revision clinics and consistent check-ups from academic and sport staff) in protecting academic success [8]. Mentorship, monitoring and extra tutoring were some of the academic support services provided at ‘Nunwick High’, which are consistent with previous sport school literature [7, 8, 93, 112, 113] and recognised as essential for encouraging academic success [114]. Finally, coach support (e.g. flexibility with sport training and support around the periods of high academic stress) was highlighted to assist student athletes academic development. This result is similar to Knight and colleagues [115], who underscored the need for an athlete’s support network to consistently reinforce the importance of education and the value of maintaining a DC. Ensuring the support staff are on the same page and everyone’s expectations are aligned, eases tensions within the group and prevents the student athletes from feeling conflicted [115].

#### Performance Sport Program with Direct Sport-Related Practices, Staff and Support Services

The current study provides additional evidence of Thompson et al. [15] cross-sectional study, demonstrating that student athletes will improve their all-round sport performance across an academic year and this change may be facilitated by a multi-disciplinary sport staff, high quality facilities, fixtures, training partners and coaching staff, high frequency of training, individualised support and a positive team culture. High-quality coaches and multi-disciplinary teams (e.g. SC coaches, sports psychologists, nutritionists and physiotherapists) are raised in the wider literature as aiding talent development [116–119]. Accordingly, it seems plausible that sport-friendly school programmes should employ high-quality coaches and support sport staff to provide high-quality training programmes and sessions. However, whilst this study demonstrates the value of high-quality coaches and support staff, future research should explore how coaches achieved performance education and development in practice. Having high-level fixtures and training partners is supported by Henriksen’s research [120], which supports a culture where you foster competition between members of the same institution and challenge them externally. However, although frequent and additional training opportunities were deemed a positive in this study, future research should explore the workload of the sport-friendly school student athletes objectively and their subsequent correlation with rest, recovery and injury.

#### Lack of Organisation and Planning of Training Load

Student athletes at ‘Nunwick High’ attributed their initial hard transition partly to inadequate physical preparation. Likewise, student athletes in Andersson and Barker-Ruchti’s [80] study attributed the initial stress they experienced due to the lower level of physical training that had taken place in their previous club communities. ‘Nunwick High’ student athletes faced an immediate, intense training load (with no preseason), possibly contributing to a high November/December (T2) injury rate. Similar findings in prior research (e.g. [121]) noted increased injuries after school holidays (e.g. summer). These findings suggest that more careful consideration of return to training planning and monitoring of appropriate training loads may be warranted [122, 123]. From a fatigue, illness and injury prevention perspective, student athletes (particularly those new to a performance sport program) may benefit from a gradual, sequential increase in intensity, frequency, and volume early in the academic year. Furthermore, student athletes may benefit from support to help them prepare for and cope with the challenges and changes of moving into or transitioning through the sport-friendly school environment [81, 85].

A recurring ‘tiredness’ theme emerged among ‘Nunwick High’ student athletes, with subsequent mental and physical fatigue accumulation. Across an academic term, ‘Nunwick High’ lacked planned deloading or periodization, with no systematic high-to-low load transitions to facilitate recovery [104]. As such, the issue may not be the overall load buts its organisation and lack of external sport workload coordination [104]. Scantlebury et al. [33] highlighted that a failure to provide appropriate periods of recovery between training sessions and within programmes could lead to lowered training capacity [124, 125] or increased incidence of injury, illness and overtraining [126–128]. Furthermore, the lack of periodised planning may explain the fact that ~ 30% of student athletes had sustained an injury. To provide a sufficient stimulus for progressive overload, student athletes need be exposed to periods of high training volume and/or intensity [2, 129], reflected in the increase in physical fitness testing data. However, recovery must be implemented after periods of intensified or voluminous training to allow the athlete to dissipate fatigue, adapt and avoid maladaptive responses such as overuse injury [108]. Accordingly, in sport schools, planned high-load/low-load periods are crucial to facilitate recovery and adaptations [33, including periodised tapering or deload weeks aligned with high academic stress periods (e.g. assessments or mock exams).

#### Lack of Coordination and Program Flexibility Between Academic and Sport Timetables

Competing demands can be stressful when activities across the school timetable are insufficiently coordinated [85]. ‘Nunwick High’ lacked coordination between academic and sport timetables (e.g. fixtures scheduled throughout high academic stress periods, where student athletes missed lessons). Although some academic staff offered extra support and coaches were somewhat flexible and supportive (although may subconsciously emphasise sport within their communication with student athletes), better program planning, communication and alignment between coaches and teachers are needed. Previous research has highlighted that flexibility and planning are key to managing student athletes’ schedules [33] and alignment between coaches and teachers is crucial [84]. Consequently, coaches and teachers should adopt an athlete-centred approach, coordinating to recognise periods of high academic stress (e.g. exams and coursework deadlines) and high sport workload (e.g. competitions, finals) before adjusting schedules to ensure student athletes can manage both demands [33]. However, this may be more difficult for some sport (e.g. summer sport, such as cricket), where timetable clashes may be unavoidable. Previous research supports such integrated efforts as critical features of successful talent development environments [20, 115], alleviating tensions and helping prevent dual career demands conflict [115].

It appeared hard for practitioners within ‘Nunwick High’ to plan effective training loads, efficient recovery and sufficient academic time due to the ‘individualised chaos’ within and between studentathletes varying weekly schedules [130]. Qualitative and quantitative (95% CI) data confirmed this variability. The challenges of within and between youth-athlete variance in weekly training load has been previously shown [33, 131]. Individual needs differ based on sport, academic path and circumstances [132]. Consequently, in addition to program flexibility, sport-friendly schools may consider monitoring sport school student athletes’ varying weekly schedules, coaches/teachers should monitor student athletes’ physical and academic loads (e.g. training/work diaries), wellness (e.g. daily wellness questionnaire [133], the profile of mood states questionnaire for adolescents [134]) and recovery states (e.g. perceived recovery scale [135]) on an individual basis.

#### Because the Environment Demanded It

A clear positive impact was that student athletes’ developed life skills and attitudes applicable in and beyond sport. The requirement to take accountability and responsibility, live away from home and balance the busy schedule of sport and academics enabled student athletes to manage themselves effectively (i.e. become better at managing multiple demands) and be disciplined. However, it is also important to acknowledge the skills required to negotiate these challenges (e.g. psychological characteristics and competencies [136]). As such, there appeared to be a need for upskilling to allow student athletes to maximise their development earlier, particularly when managing their time effectively. Collins and Macnamara [136] proposed that skills development in an appropriately challenging environment is a big factor in the pursuit of ‘super-champ’ status. As such, sport-friendly schools may consider educating the student athletes with essential skills that would aid the challenges they face during their time at the sport school (e.g. time-management skills, developing coping strategies, a programme focused on understanding the most efficient way to maximise their learning) to allow them to exploit their development by understanding the most efficient way to maximize their learning and balance the issues arising from their restricted time schedules [33, 86].

### Balance Between Optimising Experience and Appropriate Challenge

It is worth noting that while student athletes encountered many challenges throughout the school year (e.g. oscillations in stress and demanding schedules), longer-term they reported largely positive impacts, potentially preparing them for the multiple demands of being a professional athlete or adult in the future. Research emphasises the value of incorporating challenges into talent development pathways (e.g., [137] and [138]). Overcoming challenges is increasingly seen as favourable for aspiring student athletes [137, 138] but developing skills to navigate these challenges (e.g. psychological characteristics and competencies) should be planned and managed too. As such, while helping manage some of the physical overloading and scheduling (e.g. to prevent harm through injury, stress and emotional/physical fatigue), helping coaches understand progressive tolerance to the stresses experienced and upskilling student athletes is clearly warranted, there may be a need for some of these challenges to develop long-term positive holistic impacts (i.e. where the immediate/short term negative impacts could have medium-longer term positive impacts). So, while potential recommendations within this study may help optimise the experience, they should be carefully considered regarding their impact on the student athletes’ development in other areas (e.g. resilience, independence and self-motivation). Consequently, future research needs to explore what short-term impacts and processes are needed for long-term positive impacts.

## Limitations and Future Research

Although the longitudinal design, mixed methods approach (triangulation), and generalised mixed modelling analysis are key strengths, it is also important to be aware of the study’s limitations. Some would argue that due to the first-hand experiences of the primary author, they already had their preconceived ideas, potentially narrowing the analytic lens of the study. However, the quantitative statistical analysis alongside the use of critical friends and frequent peer-debriefing and reflection sessions among co-authors, to minimise any potential biases [69]. Self-reported measures introduce another limitation, including the potential influence of social desirability. Moreover, different questionnaires were necessary to capture diverse impacts, potentially impacting response quality due to the questionnaire’s length [139]. However, the questionnaire was conducted in a quiet room, student athletes were allowed sufficient breaks when required and were allowed to return to the questionnaire at a later time within the same day. Furthermore, while participant concerns might not have been openly expressed in front of an institution member, the primary author's rapport with student athletes and staff fostered positive interactions, emphasising confidentiality and encouraging open, honest responses. Finally, in the academic year, term 6 was only 3 weeks long, and most upper-sixth student athletes had already left after final exams, leading to the decision to omit the online questionnaire during this term. Despite this, observational research covered the full 33 weeks, with the timeline diagram conducted at the study’s conclusion, though the lack of log diary assessment in terms 5 and 6 is a limitation.

While this study offers an initial insight into sport–school student athletes’ holistic impacts and trajectories, future research could explore this further using longitudinal methods, such as Cobley et al. [140], tracking the comprehensive development of select youth players and employing different statistical techniques such as multivariate latent growth models (e.g. [141]). Moreover, while this study provides an initial insight into how individual characteristics shape the sport school experience and its impact on the holistic development of youth athletes, further research is needed to gain a more in-depth understanding. For example, exploring additional individual characteristics like sport-by-sport analysis, age, injury status and training cycles could further enrich understanding. Finally, while preliminary discussions about potential correlations between impacts were included (e.g. academic attainment and AM), these relationships lack statistical exploration, necessitating further modelling and investigation of direct impact relationships.

## Conclusions

Overall, ‘Nunwick High’ student athletes developed positive long-term holistic impacts (i.e. academically, athletically and personally), including maintaining stable and relatively high levels of sport confidence, academic motivation, general recovery, life skills, resilience and friends, family and free time scores. Development was generally attributed to the sport school’s athletic and academic support services and personal traits of the student athletes and staff. Moreover, accountability, responsibility, independence and navigating busy schedules fostered crucial life skills. Despite positive impacts, juggling academic and sport workload posed challenges for student athletes, potentially leading to negative holistic impacts (e.g. fatigue, pressure, stress, injury and lessons missed). These issues were linked to insufficient training load build-up, communication, coordination, flexibility and planning. While addressing physical overloading and coach understanding is important, future research should evaluate other environments and explore what short-term impacts are needed for long-term positive impacts.

Additionally, individual characteristics (e.g. biological sex) influenced sport school impact. Females had lower sport confidence, higher general stress and body image concerns and less general recovery compared with males. This vulnerability warrants detailed research on female student athletes. Furthermore, engagement in external sport introduces additional time and workload commitments, prompting sport schools to collaborate with broader sporting partners to harmonise student athletes’ training schedules and create coordinated athlete-focused training plans and goals. In summary, these findings demonstrate the complex nature of combining education and sport commitments and how sport schools should manage, monitor and evaluate the features of their programme to maximise the holistic impacts of sport–school student athletes.

## Declarations

### Availability of Data and Materials

The datasets generated during and/or analysed during the current study are available from the corresponding author on reasonable request.
